# Evaluation of a New Simplified Approach for Upper Superficial Impacted Third Molar Extraction—A Retrospective Split-Mouth Study

**DOI:** 10.3390/dj12060184

**Published:** 2024-06-17

**Authors:** Alberto Materni, Karol Alí Apaza Alccayhuaman, Alberto Maltagliati, Daniele Botticelli, Stefano Benedicenti

**Affiliations:** 1 Department of Surgical and Diagnostic Sciences (DISC), University of Genoa, 16132 Genoa, Italy; materni@maternipiano.it (A.M.); maltagliati@hotmail.com (A.M.); benedicenti@unige.it (S.B.); 2 Department of Oral Biology, University Clinic of Dentistry, Medical University of Vienna, 1090 Vienna, Austria; caroline7_k@hotmail.com; 3 ARDEC Academy, 47923 Rimini, Italy

**Keywords:** cyanoacrylate, flap design, glue, impacted tooth, maxillofacial surgery, oral surgery, third molar extraction

## Abstract

Background: A new access technique was developed to reduce postoperative adverse events after the extraction of impacted maxillary wisdom teeth. Hence, this study aimed to assess the occurrence of adverse events after the extraction of maxillary impacted wisdom teeth using a traditional access (TA) or a new technique (NT). Methods: Two different surgical incision designs were used for bilateral wisdom tooth extractions in 30 patients. The traditional incision was performed distal to the second molar in the center of the tuberosity, followed by a buccal releasing incision. After the tooth extraction, the wound was secured by sutures. The new technique consists of an oblique incision from the distal palatal aspect of the tuberosity towards the buccal aspect of the second molar. After the tooth extraction, cyanoacrylate glue was used on the wound. Results: Lower pain was reported by patients at the site treated with the new technique (*p* < 0.01). Edema, postsurgical bleeding, and hematoma were similar in both groups. The surgical time was shorter for the new technique (*p* < 0.01). Conclusions: The new technique applied for the extraction of impacted maxillary wisdom teeth reduced postsurgical pain and the duration of surgery.

## 1. Introduction

The removal of impacted third molars (Ms3) is one of the most common surgical interventions performed in dentistry [1]. In Norway alone, approximately 75,000 Ms3 are removed annually, often by oral surgeons [2,3]. Impacted Ms3 may necessitate a removal procedure that frequently involves a surgical flap, ostectomy, and occasionally odontotomy, as well as soft tissue suturing. Unfortunately, the manipulation of both soft and hard tissues can cause post-surgical swelling and pain, postoperative bleeding, hematomas, oral antral communications, reduced masticatory ability, trismus, and periodontal diseases at the distal aspect of the maxillary second molar (M2) [1,4,5,6,7]. The surgical M3 removal procedure employed has been found to have conflicting results, with varying outcomes in adjacent M2 [5,8]. Despite the existence of multiple standards, there are conflicting data on the effect of the surgical removal of Ms3, with differing outcomes for postoperative reinjury [5]. It is evident from this context that surgeons are still struggling to manage the optimal approach of impacted Ms3 and predict the postoperative complications [9]. Among the complications bear importance mandibular fractures [10], lesions to the inferior alveolar nerve [11,12,13], and alveolar osteitis [14,15,16]. Methods have been introduced aiming to control complications, such as drainage [17], or the application of platelet-rich fibrin [18], gauzes soaked with cortisone [19], or ozone [20]. Surgery was also implemented with the use of an Er:YAG Laser [21] and coronectomy to avoid inferior alveolar nerve lesions [22].

Nevertheless, maxillary wisdom tooth removal is normally considered not to be a very demanding type of surgery. This is because most of the time in that region, only the soft trabecular bone has to be removed to reach the impacted wisdom tooth. Therefore, no rotary instruments are used, but only hand instruments such as thin levers and no odontotomy are performed. As stated earlier, this situation is a common occurrence, but it is not a universal axiom. It is not difficult to describe some of the constant difficulties and complications, such as the difficulty in accessing that region, which affects the visibility. The more the patient opens their mouth, the more the coronoid process, together with the surrounding soft tissues and mussels, covers the wisdom region. Therefore, to access the surgical area with surgical instruments like a scalpel, elevator, and levers, upper wisdom tooth extractions are usually performed through a partially closed mouth. Mainly because of this reason, the surgeon’s touch-sensitive abilities often allow them to extract the wisdom teeth without seeing them beforehand. The operator draws an incision line with the scalpel, elevates a full-thickness flap, and then enters the region with an elevator trying to achieve the cervical area of the impacted wisdom tooth distally to the second molar only through tactile sensitivity. Then, by making lever movements, the tooth is dislocated distobuccally out of the bone. All these surgical semi-blinded maneuvers might be difficult to perform.

One of the most common adverse events after surgery for a mandibular impacted wisdom tooth is swelling, accompanied by various degrees of pain. The most accredited hypothesis for this side effect can be attributed to full-thickness flap elevation to expose and remove the retained tooth [23,24].

In the typical procedure for the extraction of the maxillary impacted wisdom tooth, the surgery ends by repositioning the elevated flap to its original position and maintaining it through a surgical suture. Sutures stabilize the mobilized flap in its original position, guarantee proper hemostasis, and close possible oral antral communication that might occur after the extraction when the upper wisdom teeth are in direct contact with the maxillary sinus. Based on the previously described flap for removing mandibular impacted wisdom teeth [23,24], a new flap design was created to remove maxillary impacted wisdom teeth. The extension of bone exposure was reduced by this flap, which could possibly reduce the negative effects of postoperative swelling and pain. Hence, this study aimed to assess the occurrence of adverse events after the extraction of maxillary-impacted wisdom teeth using a traditional access (TA) or a new technique (NT).

## 2. Materials and Methods

### 2.1. Ethical Statements

This study followed the Declaration of Helsinki regarding medical protocol and ethics, and the CERA (Comitato Etico per la Ricerca di Ateneo) of the University of Genoa approved the study on 16 November 2023 (protocol #2023/79). Written informed consent was obtained from all the patients who voluntarily agreed to undergo the procedure and were willing to return for evaluation at regular intervals. Signed patient releases were obtained for imaging use.

### 2.2. Study Design

In this retrospective split-mouth study, we selected a series of documented cases from the Department of Surgical and Diagnostic Sciences (DISC) database at the University of Genoa in Italy. Each case involved a patient who underwent two different incision techniques for the extraction of upper superficial impacted third molars. The traditional technique (TA; control) was characterized by a standard incision made in the middle of the crest of the maxillary tuber, full-thickness flap elevation, and wound closure with sutures. In contrast, the new technique (NT; test) involved a single oblique incision, no flap and periosteum elevation, and wound closure with glue. Despite the retrospective nature of our study, we adhered. In all bilateral cases, a simplified method of randomization, i.e., tossing a coin, was adopted.

### 2.3. Inclusion and Exclusion Criteria

All selected patients were treated for bilateral maxillary third molar extractions (Ms3). To be included in the study sample, patients had to meet the following inclusion criteria, as evaluated on the database of the Department of Surgical and Diagnostic Sciences (DISC): (1) bilateral extraction of impacted upper Ms3; (2) availability of clinical and radiographic data. The exclusion criteria were as follows: (1) monolateral extraction of impacted upper Ms3; (2) lack of clinical or radiographic data sufficient to harvest all necessary information needed for the study. Moreover, no extractions of upper impacted third molars were performed in patients with compromised health conditions.

The case selection was rigorous, seeking only cases with impacted teeth with comparable position, depth, and presumed buccolingual impaction on both sides. All evaluations were made exclusively using panoramic radiographies.

### 2.4. Sample Size Calculation

The sample calculation was based on the results of a previous study on the extraction of mandibular wisdom teeth performed by the same research group [23]. A power of 0.95 and α error probability of 0.05, with a correlation between groups of 1.00 for edema, and 1.040 for pain, yielded a sample of 16 and 15, respectively. However, given that the extraction of maxillary wisdom teeth generally results in a lower degree of edema and pain compared to the mandibular wisdom teeth, it was decided to increase the sample to about double.

### 2.5. Presurgical Evaluations and Treatments

The upper superficial impacted third molar extractions was performed in patients that asked for this treatment. The reasons were mostly for orthodontic treatment and rarely for pain. Before scheduling the patient for surgery, adequate professional hygiene and a review of oral maintenance were performed to achieve optimal plaque control with an average F.M.P.S. of < 20% in the study population. The participants were radiologically screened using an orthopanoramic radiograph (Planmeca ProMax^®^ with a one-shot cephalostat; Helsinki, Finland).

### 2.6. Surgical Procedures

All surgeries were performed by the same surgeon (A.Mat.) and all patients underwent bilateral surgery on the same visit. Two cartridges of local anesthetic (2 × 1.8 mL of articaine 4% with adrenaline 1:100.000) were injected per side, buccal and palatal. The surgeon always started on the right side of the patient at tooth 1.8 and as second on the left side at tooth 2.8.

**Figure 1:**
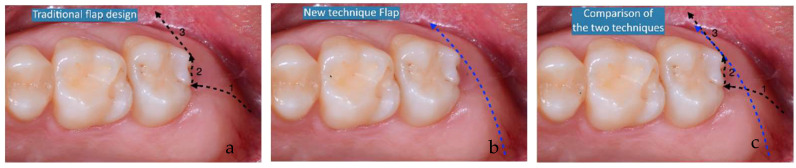
Incision lines illustrating the: (**a**) traditional access (TA); (**b**) new technique (NT); (**c**) Superposed incisions. The numbers indicate the sequence of incisions. The arrows indicate the direction of the incisions.

The two surgical techniques were compared (Figure 1a–c).

**Figure 2:**
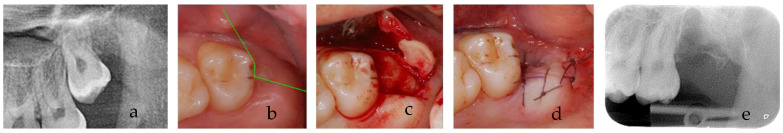
(**a**) radiographic image of a left impacted maxillary wisdom tooth; (**b**) incision lines illustrating the traditional access; (**c**) flap opened; (**d**) closure with sutures of the wound after extraction; (**e**) radiographic evaluation after extraction.

The traditional technique (TA) for the extraction of an upper impacted third molar (Figure 2a) consists of a straight incision, beginning in the middle of the crest of the tuber maxillae and progressing forward to the middle of the distal surface of M2 (Figure 2b).

In this region, the second incision began in the distal sulcus of M2 and proceeded buccally intrasulcular of the M2 ending at the distobuccal edge of M2. From this point, a releasing incision through the buccal attached the gingiva over the mucogingival line described the third and last incisions. All the incisions were made using a 15c Swann-Morton blade (Figure 2c) and a triangular full-thickness flap, that is including the periosteum, was elevated with a Prichard elevator (3 Prichard periosteal PPR36, Hu-Friedy Mfg. Co., Chicago, IL, USA). The wisdom tooth area was reachable using a straight 3 mm lever (Luxator Periotome L3S TiN Directa, Lecce, Italy), which was gently pushed between the distal surface of M2 and the mesial surface of M3 to the cementum enamel junction of the impacted M3. When the surgeon felt that position, he began to rotate the lever distobuccal in order to extract the impacted M3. After the removal of M3 with the help of a 3 mm Lucas bone curette, the residual follicle of M3 was gently removed, and the residual thin, poorly attached bony walls of the empty alveolus were removed. A 5 mL sterile physiological solution wash was performed directly inside the wound using a single-use syringe. Subsequently, a resorbable bovine collagen sponge (hemocollagen; SEPTODONT, 58 rue du Pont de Creteil, Saint Maur des Fosses, France) was inserted into the wound directly in the superficial portion of the alveolus to stabilize the coagulum. The surgery ended by repositioning the elevated buccal flap in its original position and was secured using a monofilament synthetic absorbable poliglecaprone 4/0 USP surgical PGCL suture (OMNIA S.p.A., Fidenza, Italy). Single sutures were used to close the first distal incision and the buccal area to close the third releasing incision (Figure 2d). An intraoral X-ray was only taken in case of uncertainty regarding the complete extraction of the element or for the evaluation of possible fractures of the maxillary tuberosity (Figure 2e).

**Figure 3:**
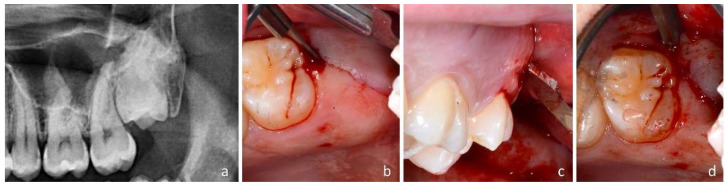
(**a**) radiographic image of a left impacted maxillary wisdom tooth; (**b**) single oblique incision; (**c**) incision extending to the buccal aspect; (**d**) thin straight lever inserted between M2 and M3.

The newly proposed technique (NT) for impacted maxillary wisdom teeth (Figure 3a) consisted of a single oblique incision that began in the keratinized mucosa on the distal palatal side of the tuber maxillae and crossed from the distopalatal side to the mesiobuccal side (Figure 3b).

The 15c blade entered the gingival sulcus of M2 at its distal edge and continued its oblique path through the buccal attached gingiva, reaching the mucogingival line at the buccal side of M2 (Figure 3c). This incision was made straight from the distopalatal portion of the tuber maxilla to the buccal mucogingival line of the buccal surface of M2. No flap was elevated so that the periosteum was not elevated but left adherent to the bone. Through the performed incision, at the distobuccal edge of the M2, a thin 3 mm straight lever (Luxator Periotome L3S TiN Directa, Lecce, Italy) was inserted in the incision entering the tissues in contact with the distal surface of the M2. The lever was gently pushed inside the incision to reach the crown of M3 and subsequently between the crown of M3 and the distal surface of M2 (Figure 3d).

**Figure 4:**
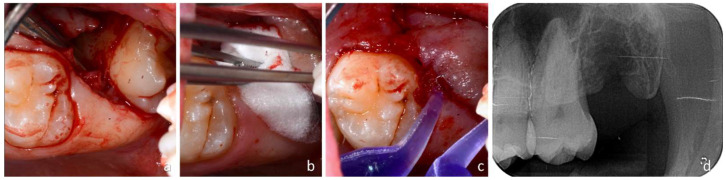
(**a**) Dislocation of the wisdom tooth; (**b**) placement of a collagen sponge in the alveolus; (**c**) glue to fix the wound; (**d**) radiographic evaluation after extraction.

When, by gently moving the lever, it was deemed that the cementum-enamel junction of M3 had been reached, a rotation of the lever was started to push M3 out of the wound towards the oral cavity. Soft tissues were cut through an oblique incision, and the wound was spread apart by the emerging tooth during extraction (Figure 4a).

Subsequently, a revision of the alveolus was performed using a 3 mm Lucas bone curette to remove the follicle of M3 and possible thin bone walls that were fractured during the extraction of M3. Subsequently, a wash was performed with 5 mL sterile physiological solution directly inside the alveolus using a disposable syringe. An absorbable bovine collagen sponge (Hemocollagene, SEPTODONT; Rue du Pont de Creteil 94100, Saint Maur des Fosses, France) was inserted into the wound directly in the superficial portion of the socket to stabilize the clot (Figure 4b). As this surgical technique did not involve any flap lifting, the wound was closed using surgical glue (PeriAcryl^®^ 90HV Cyanoacrylate Oral scription; GluStitch Inc. 307–7188 Progress Way—Delta, British Columbia V4G 1M6, Canada) directly on the wound (Figure 4c). A gentle stream of sterile saline solution was used to immediately set the glue, and an X-ray was taken to evaluate the complete extraction of the roots and possible complications, such as fractures of the tuberosity (Figure 4d).

### 2.7. Clinical Evaluations

Patient sex and age were recorded. The following clinical parameters were evaluated and recorded: pocket probing depth distal (PPD-D) and distovestibular (PPD-DV) at the second molar.

The operative parameters evaluated were subjective difficulty encountered during extraction (0, straight through; 1, moments of uncertainty); bleeding within a few minutes after the end of the surgical procedure (0, no bleeding; 1, light bleeding; 2, heavy bleeding); and time for extraction from the first incision to when the last stitch was cut or when the gentle physiological washing of the glue to make it hardened quickly was finished. The bleeding considered “normal” was that which is typical of an incision in a flap (no bleeding); when hemostasis was achieved solely through compressive maneuvers with gauze for at least two minutes, it was considered “light bleeding”; if it became necessary to use drugs such as tranexamic acid to achieve hemostasis, then we referred to it as “heavy bleeding”.

Telephone calls were made the following day. At the 1-week visit, a VAS was used for the following evaluations: home bleeding (0, no bleeding; 1, light bleeding; 2, heavy bleeding), pain (0, no pain; 1, bearable pain; 2, significant pain; 3, unbearable pain), edema (0, absent; 1, light-perceptible; 2, evident; 3, extreme-deformative), and hematoma (0, absent; 1, less than 2 cm in diameter; 2, more than 2 cm in diameter). Grade 2 home bleeding, and grade 3 pain and edema were considered complications.

Wound closure was evaluated after 4 and 8 weeks (0, closure; 1, no complete closure). At the 8-week visit, pocket probing depth distal (PPD-D) and distovestibular (PPD-DV) at the second molar and complaints (0, no complaints; 1, complaints, reporting type, and degree) were assessed.

### 2.8. Experimental Outcomes and Statistical Methods

The primary predictor variable was the surgical technique employed, encompassing incision, periosteum elevation, and closure methods. The primary outcomes variables were postoperative pain and edema. The secondary outcome variables included bleeding, hematoma, and surgery time. The values obtained are expressed as mean ± standard deviation. The Shapiro-Wilk test was used to determine the normality of data and, according to the results, the differences between the test and control sides were evaluated using a paired *t*-test or a Wilcoxon matched-pairs signed rank test.

## 3. Results

### 3.1. Demographic and Presurgical-Surgical Data

**Figure 5:**
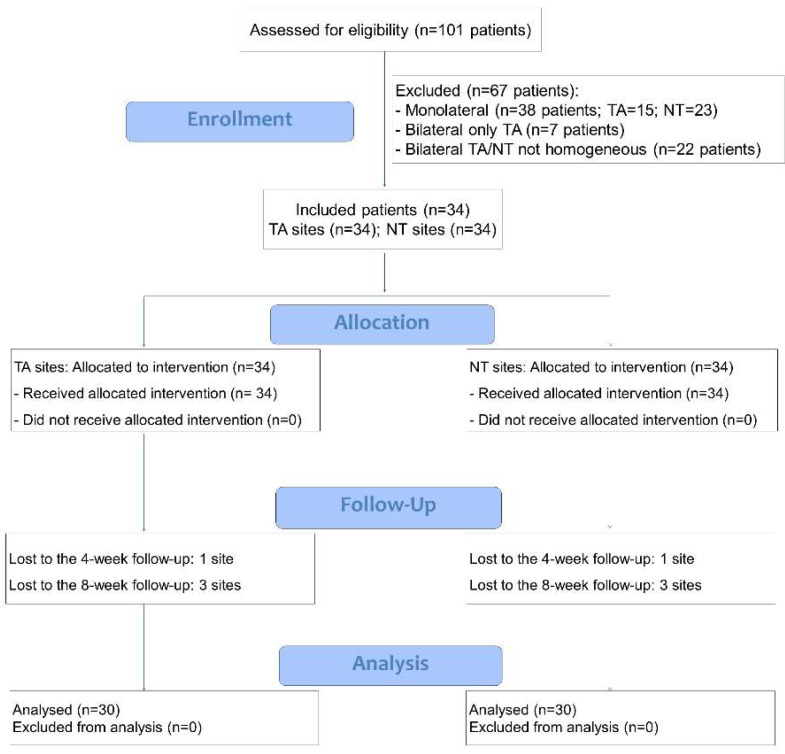
Flow chart.

One hundred and sixty-four upper impacted third molars were extracted in one hundred and one patients at the Department of Surgical and Diagnostic Sciences in a period included between 1 April 2022 and 31 March 2023. Sixty-three patients received a bilateral extraction. However, only thirty-four patients presented teeth with homogeneity in position, depth, and presumed buccolingual impaction and were eligible for the treatment (See the flow chart in Figure 5). However, 4 patients missed the follow-up and were excluded from the analysis so that thirty patients were included in the analysis. The mean age was 32.1 ± 10.8 years, 14 were females, and 16 were males. The initial probing depth in the TA group was PPD D = 3.9 ± 0.7 mm and PPD DV 2.5 ± 0.5 mm. In the NT group, the respective measures were 4.1 ± 0.9 and 2.5 ± 0.6 (*p* = 0.109 and *p* > 0.999, respectively).

### 3.2. Evaluation at the Surgery

The mean time used for all tooth extractions was 07:03′ ± 02:22′ in the NT group and 11:01′ ± 02:17′ in the TA group (*p* < 0.0001). Difficulties during extraction were found in three cases in the NT group, with a mean time of 11:06′ ± 02:22′, and 3 cases in the TA group, with a mean time of 15:00′ ± 02:53′. The difficulty encountered in all three cases TA and NT was solely related to the mobilization of the impacted element using Bein’s lever. Bleeding was not observed at the end of the surgical procedure. 

### 3.3. Evaluation at 1-Week of Healing

**Figure 6:**
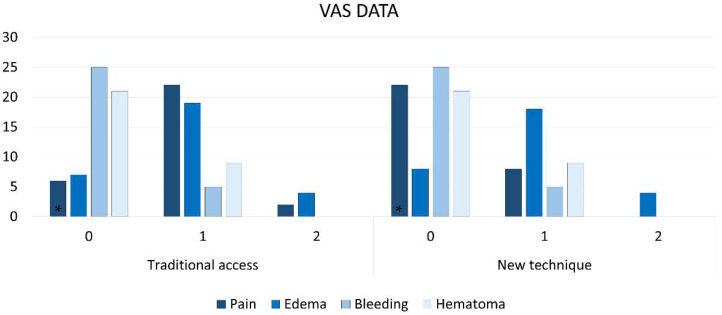
Number of patients presenting various grades of pain, edema, bleeding, and hematoma. Pain (0, no pain; 1, bearable pain; 2, significant pain; 3, unbearable pain), edema (0, absent; 1, light; 2, evident; 3, extreme), home bleeding (0, no bleeding; 1, light bleeding; 2, heavy bleeding), and hematoma (0, absent; 1, less than 2 cm in diameter; 2, more than 2 cm in diameter). * = *p* < 0.05.

No major complications were registered. No pain was reported by 22 patients of the NT group, and only by 6 patients of the TA group (Figure 6); 2 patients in group TA reported significant pain while all remaining patients of both groups reported bearable pain (*p* < 0.0001 between groups). The maximum grade of edema was 2 (evident), reported by four patients in both groups, while in most cases, light-perceptible edema was reported (*p* > 0.999 between groups). No home bleeding was reported by 25 patients in both groups while five patients in each group reported a grade 1. Hematomas were reported by 9 patients each group (grade 1; *p* > 0.999).

### 3.4. Evaluation at the Follow-Ups

After 4 weeks, 25 wounds were closed in the NT group and 17 in the TA group (*p* < 0.01). However, after 6 weeks, all the wounds were closed in both groups (Figure 4d). At 8 weeks, no complaints were reported by any of the patients. The PPD D and PPD DV in the NT group were 3.4 ± 0.5 mm and 2.4 ± 0.6 mm, respectively. The differences between the preoperative and 8-week postoperative probing depth yielded *p* < 0.01 for PPD D and *p* = 0.500 for PPD DV. In the TA group, the respective probing depths were 3.6 ± 0.7 mm and 2.5 ± 0.5 mm (*p* = 0.100 and *p* > 0.999, respectively).

## 4. Discussion

This study aimed to evaluate the postoperative adverse events after maxillary-impacted wisdom tooth extraction using traditional or modified access. The two techniques differ in terms of incision access, flap elevation, and wound closure. Traditional incision includes a straight incision in the middle of the tuber and a releasing incision in the distal aspect of the second molar. A full-thickness flap including the periosteum is elevated. The modified access includes only an oblique incision from the distal palatal side of the tuber to the distal buccal aspect of the second molar, crossing the attached buccal gingiva to the buccal mucosa. The peculiarity of this modified incision is that, besides allowing access to the impacted tooth with levers, it might be dislocated distobuccally like a trapdoor without elevating the periosteum. Without flap elevation, the elasticity of the flap allows the tooth to be enucleated, and owing to its elastic memory, it regresses to its original position. Therefore, after the placement of a collagen sponge, no sutures are needed to secure the flaps, and only cyanoacrylate glue can be applied.

The modified flap presented several advantages. The pain reported by the patients was lower in the modified access group than in the traditional access group (*p* < 0.0001 between groups), even though only two patients in the TA group reported significant pain. No differences were reported in edema. However, the time of surgery was significantly lower, and the wound closure was faster for the NT compared to the TA technique.

Flap design has assumed particular importance in recent years, especially for the extraction of impacted mandibular wisdom teeth. Attention has been paid to improving periodontal healing in the second molar and wound closure [25,26,27,28]. However, in these studies, no significant advantages were observed for postoperative pain or edema.

Recently, a modification of the access for the extraction of impacted third mandibular molars has been suggested. Traditional access with flap elevation was compared with complete removal of the oral mucosa above the impacted tooth, which is described as a flapless surgical approach (FSA). A lower degree of pain and edema was observed in the modified FSA approach than in the traditional approach [23]. A further improvement was subsequently suggested by the same group [24]. In an RCT study, the FSA technique was compared with a new technique called “single incision access” (SIA), which only included a small semilunar incision above the buccal profile of the impacted tooth. From this “small” access, the wisdom tooth could be extracted. The SIA approach produced results similar to those of the FSA in terms of postoperative pain and edema; however, the recovery of the treated region was faster.

This confirmed that the management of soft tissues is extremely important for postsurgical patient comfort. Applying similar care for soft tissue management for the extraction of maxillary impacted wisdom teeth, the postsurgical period of the patients also improved in quality compared with the traditional technique. After 4 weeks of healing, 25 wounds were closed in the modified flap group and 17 in the traditional flap group (*p* < 0.01). Moreover, the modified surgical procedure used in the present study was faster than that of the traditional flap approach, that is, 7 min compared with 11 min, which is also in favor of the modified access. Some may argue that the use of glue significantly affected the time calculation. However, it is important to note that no flaps were elevated in the NT group, allowing the periosteum to remain adhered to the bone. This resulted in the modified flap tending to regress to its original position, thereby favoring the use of glue over sutures. In contrast, the traditional access (TA), which involved full-thickness flaps and periosteum elevation, did not allow for a secure wound closure using glue, and sutures had to be used to secure the wounds. This implies that the variables to be considered should not be two—incision and sutures—but rather only one, “the technique”, that includes flap and closure. Finally, the difficulties in tooth extraction were similar in both groups, indicating that the new flap did not complicate the surgical procedure.

The present study has limitations. Even though it was performed with accuracy in collecting data and taking care of patients, it was not initially meant to be a randomized clinical trial. The intention was only to evaluating the effectiveness of the NT approach in comparison to the standard access applying a retrospective approach. Clinical cases were added overtime and finally, given the goodness of the technique, it was decided to make the surgical procedure known for clinical use. The selection of the cases among one hundred and sixty-four extractions in one hundred and one patients was rigorous and difficult, because it was decided to select only cases with bilateral impacted upper third molars with comparable position, depth, and presumed buccolingual impaction. The sample was limited but sufficient in size to disclose some differences in terms of pain, healing pace, and time for the surgery.

## 5. Conclusions

In conclusion, the implementation of a simplified incision did not complicate the surgical procedure; rather, it facilitated the use of adhesive agents instead of sutures, resulting in significant time savings, reduced pain, and faster wound healing. These results highlight the effectiveness and practicality of the new technique, suggesting a promising approach for enhancing outcomes and efficiency in impacted maxillary wisdom teeth extraction procedures. However, randomized controlled trials with larger sample sizes are needed to confirm the outcomes reported in this study.

## Data Availability

Data is available under reasonable request.
